# Time Evolution of the Excimer State of a Conjugated Polymer Laser

**DOI:** 10.3390/polym9120648

**Published:** 2017-11-27

**Authors:** Wafa Musa Mujamammi, Saradh Prasad, Mohamad Saleh AlSalhi, Vadivel Masilamani

**Affiliations:** 1 Department of Physics and Astronomy, College of Science, King Saud University, Riyadh 11451, Saudi Arabia; walmujammi@ksu.edu.sa (W.M.M.); saradprasad@gmail.com (S.P.); malsalhy@gmail.com (M.S.A.); 2 Research Chair on Laser Diagnosis of Cancers, College of Science, King Saud University, Riyadh 11451, Saudi Arabia

**Keywords:** conjugated polymer (PFO), UV laser, amplified spontaneous emission spectra (ASE), time resolved study

## Abstract

An excited dimer is an important complex formed in nano- or pico-second time scales in many photophysics and photochemistry applications. The spectral and temporal profile of the excimer state of a laser from a new conjugated polymer, namely, poly (9,9-dioctylfluorenyl-2,7-diyl) (PFO), under several concentrations in benzene were investigated. These solutions were optically pumped by intense pulsed third-harmonic Nd:YAG laser (355-nm) to obtain the amplified spontaneous emission (ASE) spectra of a monomer and an excimer with bandwidths of 6 and 7 nm, respectively. The monomer and excimer ASEs were dependent on the PFO concentration, pump power, and temperature. Employing a sophisticated picosecond spectrometer, the time evolution of the excimer state of this polymer, which is over 400 ps, can be monitored.

## 1. Introduction

Of all the four class of laser media (solid, liquid, gas, and plasma), liquid solutions (such as organic dye dissolved in ethanol) have certain unique advantages and are often called the ‘pipe dream’ of laser physicists [1]. This is because the liquids are essentially self-curing and could be circulated to enhance overall working time durability. Secondly, they could be mode locked to produce a train of picosecond pulses or made to work in continuous wave (CW) mode. Above all, most of them are tunable over the entire visible region of the spectrum with the additional advantage of working in superradiant mode.

Mostly, organic dyes, such as coumarins or rhodamines, were popular for a few decades, until the conjugated polymer (CP) came into competition, often supplementing or complementing the conventional dyes. This is because CPs have large Stokes shift by 100 nm, or more, which would reduce the reabsorption loss and lead to potential high energy application by increasing the concentration of active species in a number of reports that for conjugated polymer like MEH-PPV, photochemical stability is many times better than conventional laser dyes [2].

However, CW conjugated polymer laser (CPL) and solid state CPL have not been realized baring a few preliminary reports [3]. To achieve such versatility, it is important to understand the behavior of these molecules in excited state under population inversion (PI) condition. The main objective of this paper is to gain insight into the interaction between excited molecules on nano and picosecond (ns and ps) time scales by observing through their laser-induced fluorescence and optical gain spectra. In the former the PI is absent; but in the latter PI is significantly higher. To study the excited state dynamics (ns and ps time scale) of the molecules under high population inversion conditions, ASE spectroscopy is perhaps the best-cited technique as it gives direct correlation. Another related technique, called the pump-probe method, has been efficiently employed for ps photochemistry by Zewail et al. [4], however, only for our experimental condition is PI essential.

In our previous papers we have reported that the dimer could be due to the weak interaction between ground state molecules [5]. Another group reported the formation of a β-phase of PFO [6], i.e., the polymer chain conformation changes from linear (α) to spiral or coil (β) phase. However, the dynamics of PFO was not shown in time domain.

In some types of molecules, the first excited singlet state interacts with the unexcited molecules of the same species and produces an excited-state dimer (called excimer). This type of transient species is stable only in the electronically excited state, but dissociates in the ground state [7,8]. Fluorescence from excimer in concentrated solutions of pyrene was reported by Kasper and Förster. They found dramatic changes in fluorescence spectra, but for the same concentrations, the profile of absorption spectrum remained unaltered. At high concentration, the new blue fluorescence band was formed due to excimer of pyrene molecules [9,10]. This subject was later studied by Birks et al. in 1964 in 1:2-benzanthracene derivatives and pyrene solutions [11]. Martinho et al. studied the fluorescence quenching-rate constants of a pyrene excimer using CH_3_I in methyl-cyclohexane at several temperatures [12]. The excimer fluorescence spectrum could be produced in highly concentrated solutions of polymers; however, producing it from the gas or solid state is difficult.

The so-called bi-molecular excited states can be generated either optically or electrically by electron-hole recombination, i.e., they appear in both photoluminescence (PL) and electroluminescence (EL). An interesting combination of these two emissions has recently been observed and called electro-photoluminescence (EL–PL) [13]. Such conjugated molecule have shown interesting nonlinear optical properties [14,15,16] and verity of photoelectronic devices [17], such as in OLEDs [18,19], sensors [20], switches [21], and lasers [22].

Despite such extensive works, very few of these studies could produce a laser in a liquid-solution state. A few years ago, the first excimer laser from conjugated poly(2-methoxy-5-(2 ethylhexyloxy)-1,4-phenylenevinylene) (MEH-PPV) in a liquid state was reported by Masilamani et al. [2]. Subsequently, a variety of modifications were demonstrated [23,24], and the current paper is the logical extension of the time-resolved laser spectral features of the excimer state of a conjugated polymer (CP).

## 2. Experimental

**Figure 1:**
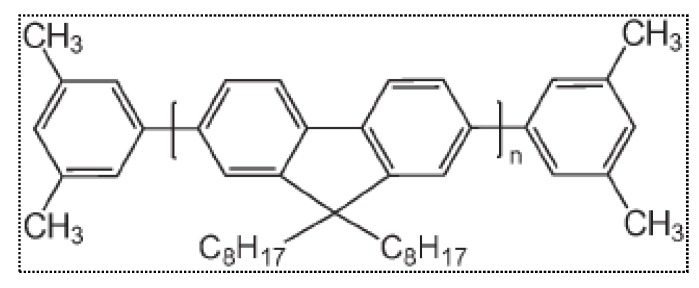
Molecular structure of the PFO polymer.

The polymer poly (9,9-dioctylfluorenyl-2,7-diyl) (PFO) was purchased from American Dye Source (Quebec, QC, Canada) and used as received. This polymer is a macromolecule with a molecular mass of 120,000. The purity of the sample was examined using thin-layer chromatography, and the results showed that the purity was more than 98%. The molecular structure of PFO, shown in Figure 1, was dissolved in benzene (spectroscopic grade with a purity of 99.8%) under a wide range of concentrations.

The absorption and fluorescence spectra of PFO in benzene under different concentrations were recorded. The spectra of the solutions were obtained using a small quartz cuvette with dimensions of 1 cm× 1 cm × 4 cm and an optical path length of 1 cm. The absorption spectra were recorded at room temperature using a Perkin Elmer lambda 950 spectrophotometer (Perkin Elmer, Llantrisant, UK) over the range from 330 to 550 mm, and the fluorescence spectra were recorded using a Perkin Elmer LS 55 spectrofluorometer (Perkin Elmer, Llantrisant, UK) in the range from 400 to 500 nm.

The excitation source was a 10 ns pulse width from third-harmonic (355 nm) of Nd:YAG laser. The samples in a four-side polished quartz cell were transverse excited by the pump laser beam focused using a quartz cylindrical lens with a focal length of 5 cm. At best combination of pump power and PFO concentration, we could get an amplified spontaneous emission (ASE) beam that came out as a cone of light. This was collected by a fiber optical cable and fed to a spectrograph, (Acton SP-2360 Imaging 98 Spectrograph, Princeton Instruments, Trenton, NJ, USA) attached to an emCCD camera (Princeton Instruments, Trenton, NJ, USA, PI-99 MAX4: 1024 × 256, 25-mm-RB intensified camera system) to obtain a three-dimensional (3D) display of the spectral and temporal features of the ASE [25].

## 3. Results and Discussion

The absorption and fluorescence properties of PFO in different solvent has been studied, and it was found that this PFO in benzene has twice better fluorescence quantum yield than any other solvent studied [5]. Due to this, the threshold pump energy was significantly lower in benzene solution and perhaps because of this the output was high.

**Figure 2:**
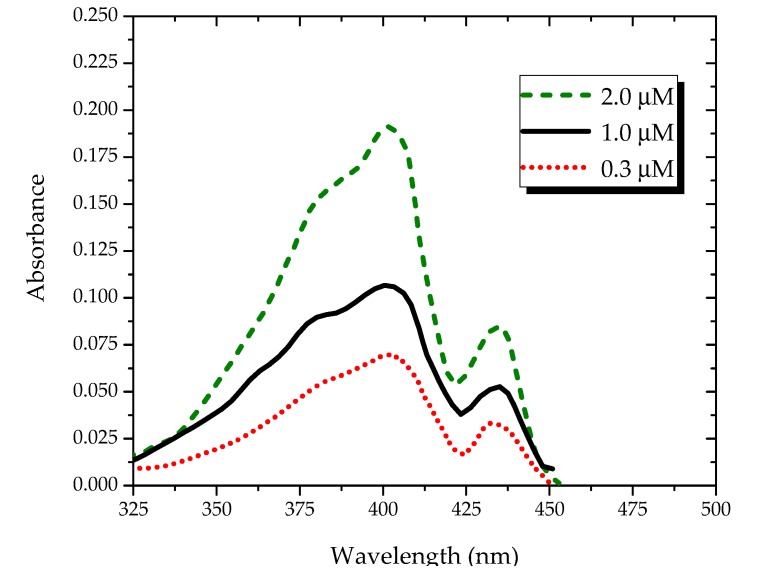
Absorption spectra of the PFO in benzene under different concentrations.

Figure 2 shows the absorption spectra of the PFO in benzene under different concentrations. It shows two absorption peaks at 400 and 437 nm. By increasing the concentration, the optical density of the peaks increased, as shown in Figure 2. The peak at 437 nm could be attributed to the ground-state aggregation, which is most likely a dimer, because it increased with the concentration in relative proportion to the peak at 400 nm.

**Figure 3:**
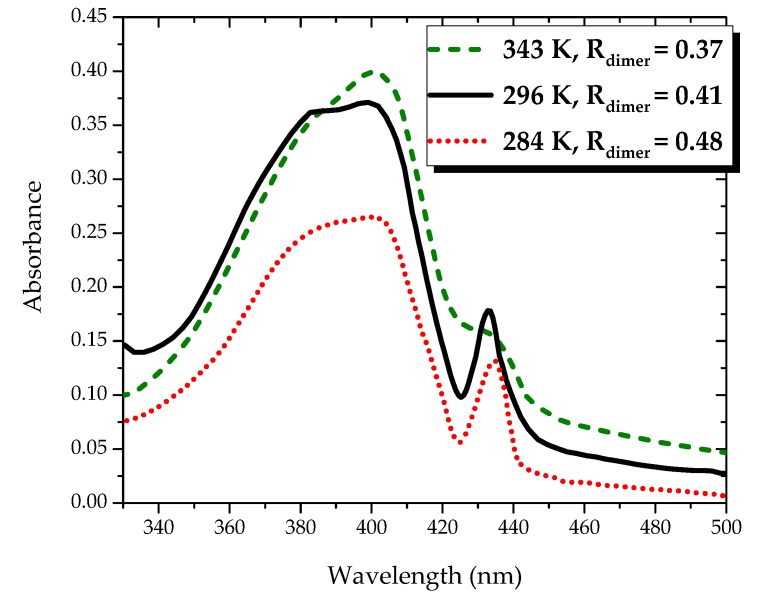
Absorption spectra of the PFO in benzene at a concentration of 4 µM under different temperatures.

Such ground-state aggregation not only depends on the concentration, but also on the temperature. To confirm the effect of temperature on the absorption spectrum, we studied the absorption spectrum of PFO dissolved in benzene at a concentration of 4 μM under different temperatures of 296, 343 and 284 K. At room temperature, the optical density ratio between the peaks from 437 nm (dimer) to 400 nm (monomer) $R_{dimer}=\;\frac{I_{437}}{I_{400}}=0.41$, as shown in Figure 3. When the temperature increased to 343 K, the peak at 437 nm became a weakshoulder, $R_{dimer}$ was reduced to 0.35 on the other hand, when the temperature decreased to 284 K, both peaks decreased but the ratio $R_{dimer}$ increased to 0.47, this result shows a strong indication of dimer formation of PFO in benzene.

**Figure 4:**
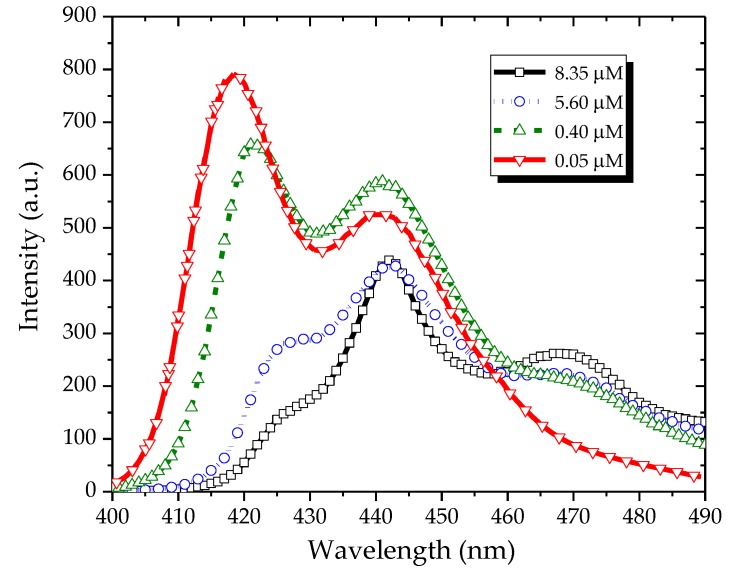
Fluorescence spectra of the PFO in benzene under different concentrations.

Figure 4 shows the fluorescence spectra of the PFO in benzene and excited at 355 nm. At a low concentration (0.05 µM), the fluorescence spectrum had three peaks: one at 422 nm and the other two at 444 and 465 nm. At a concentration of 0.4 µM, the peak at 444 nm became almost comparable with that at 422 nm, and the peak at 465 nm increased. By increasing the concentration to 5.6 µM, the intensity of the peak at 422 nm decreased, whereas the peaks at 444 and 465 nm became stronger. Under a still higher concentration (8.35 µM), the peak at 422 nm completely disappeared, that at 444 nm became weak, and that at 465 nm became dominant.

**Figure 5:**
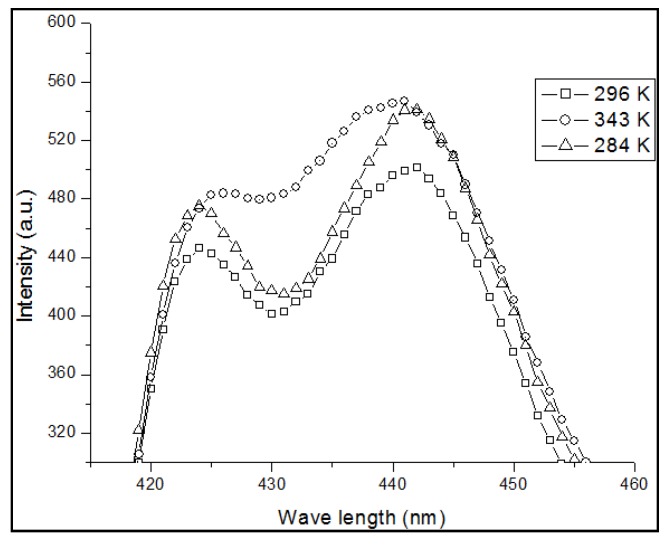
Fluorescence of the PFO in benzene at a concentration of 1.4 µM under different temperatures.

The spectral profile of the PFO in benzene at a concentration of 1.4 µM under different temperatures was studied, as shown in Figure 5. At room temperature, the ratio between the monomer and excimer was 1:1.2, and a valley existed between them. When the temperature increased to 343 K, the ratio between the monomer and excimer became 1:1.1, and the valley between the two peaks became shallow. On the other hand, at a temperature lower than room temperature (284 K), the valley became deeper, and the ratio increased to 1:1.4. All these results indicate excimer formation with an emission peak at 440 nm. A very similar trend was displayed for the emission band at 470 nm; however, this trend could be attributed to the dimer formation because a concentration- and temperature-dependent absorption peak existed at 437 nm. We also note that the monomer (390 and 420 nm in the absorption and emission processes, respectively) and the dimer (440 and 480 nm in the absorption and emission processes, respectively) had similar Stokes shift. In contrast, the excimer was manifested only in the emission (440 nm) and not in the absorption process.

**Figure 6:**
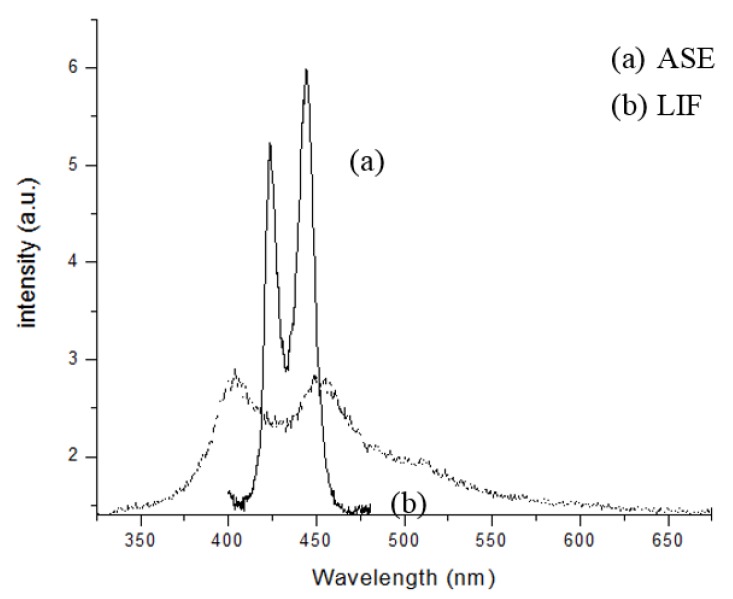
LIF and ASE spectra of the PFO in benzene.

Figure 6 shows the laser-induced fluorescence (LIF) and ASE spectra. The LIF spectrum (labeled as (b), dotted line) was for the PFO dissolved in benzene at a concentration of 0.6 µM and pump energy of 1 mJ. It displayed two peaks at 420 and 440 nm for the monomer and excimer, respectively, with a bandwidth of ~20 nm each. The ASE spectrum, (labeled as (a), solid line) was for the PFO at a concentration of 5.6 µM and pump energy of 3 mJ. The ASE peaks were approximately at 423 and 444 nm, with a narrow spectral bandwidth of 6 and 7 nm (full width at half maximum(FWHM)), respectively.

We can see that, for this concentration, the ASE at 444 nm was 15% higher than that at 423 nm. At a lower pump energy and concentration, this ratio could be reverted (not shown).

### 3.1. Time Evolution of the ASE Spectra of a PFO Laser

Several studies on the spectral features of CPs, such as the PFO and MEH-PPV have produced lasers from the monomer, dimer, excimer, and double excimer states [26]. However, no one has conducted a time-domain study on any of them. This paper presents the excited-state behavior in the sub-nanosecond time scale, which is typical of a CP host. To actually monitor an excimer, which exists only for 2 ns or less, we recorded the spectra using a picosecond camera. The formation of the excimer depends on a number of factors, but mainly on the concentration, temperature, nature of monomer, and pump power. The time scale of the monomer, excimer, and dimer states under different concentrations and pump powers is presented next.

### 3.2. Excimer Dynamics Based on the Concentration of the PFO Solution

**Figure 7:**
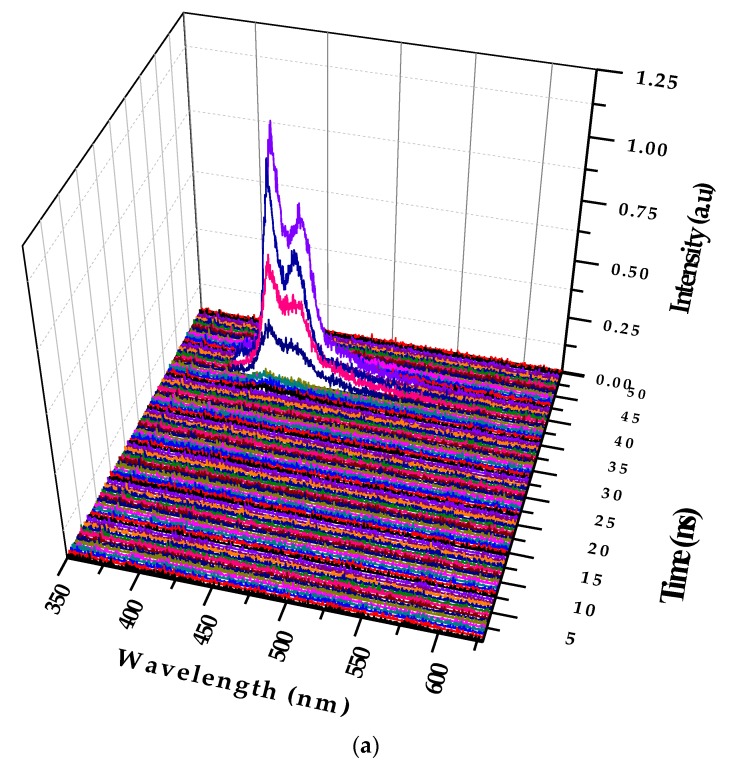

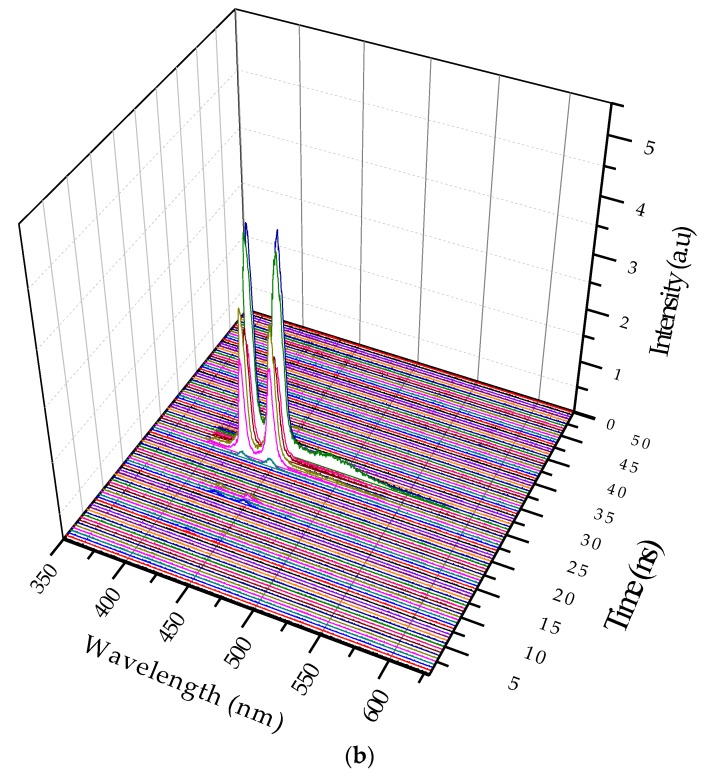
(**a**) Temporal profile of the LIF PFO at a concentration of 0.1 µM and pump power of 6 mJ (to differentiate, each frame is given different colors); (**b**) Temporal profile of PFO at a concentration of 0.6 µM and pump power of 6 mJ. After 1.5 ns of initial ASE peak (from the monomer and excimer), a new signal of approximately 480 nm occurred, which indicates the formation of the dimer for a period of 500 ps only.

The figures below show the 3D profiles of LIF and ASE with the wavelength, intensity, and time in the *XYZ* axes, respectively. The time scale for the *Z* axis is 500 ps (0.5 ns). Figure 7a shows the temporal profile of the LIF of PFO at a concentration of 0.1 µM and pump power of 6 mJ. Figure 7a shows that the peak started only after 15 ns of the trigger signal from the Q-Switch, and the spectra had two peaks attributed to the monomer and excimer. The total lifetime of the LIF was approximately 4 ns. 

Figure 7b shows the 3D ASE profile of PFO. The pump energy was kept constant (6 mJ), and the concentration of the solution was increased six times (0.6 µM). The 3D temporal profile showed two ASE peaks after 14 ns from the trigger with values of approximately 420 and 440 nm. The FWHMs were 7 and 8 nm, respectively, but after 1.5 ns, a new fluorescence band of approximately 480 nm occurred, which existed only for 500 ps, and then disappeared. This was the LIF from the dimer state of the PFO. The propagation delay of 1mlong coaxial cable is almost 5 ns, so the camera obtains the trigger signal only after 5 ns and starts acquiring data immediately. The laser pulse also travels a distance of 1.5 m before reaching the sample, taking about 5 ns (to produce ASE). The gate of the camera opens every time with a progressive delay of 0.5 ns. These operational parameters can be adjusted to suit the experiment.

### 3.3. Excimer Dynamics under Different Pump Power

**Figure 8:**
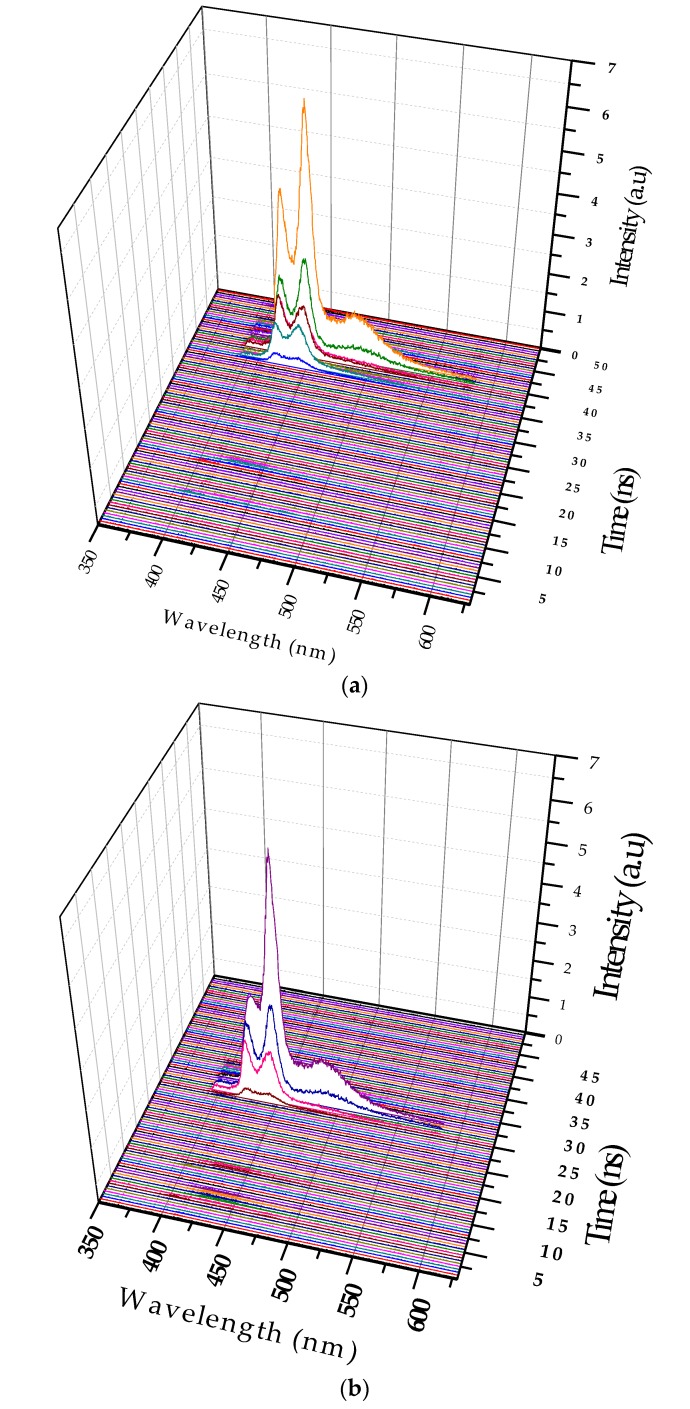
(**a**) ASE profile of the 0.8µM PFO at a pump power of 6 mJ; (**b**) Spectra under the same concentration, but with the pump energy, increased to 12 mJ.

In this case the solution concentration was increased to 0.8 µM, and the pump power was fixed at 6 mJ. Figure 8a shows two LIF peaks (at 420 and 440 nm), which evolved to ASE after approximately 5 ns; they competed in terms of intensity. Eventually, the excimer band at 440 nm became stronger. In addition, an LIF at 480 nm occurred because of the dimer. Without changing the other parameters, the pump power was increased to 12 mJ. The ASE production took less time, and the excimer formed 400 ps after the initial LIF. In other words, the increase in the pump power made the entire event occur faster; hence, the excimer formed and disintegrated, rather rapidly, as shown in Figure 8b. Note that the ASE at 440 nm due to the excimer was decisively stronger.

**Figure 9:**
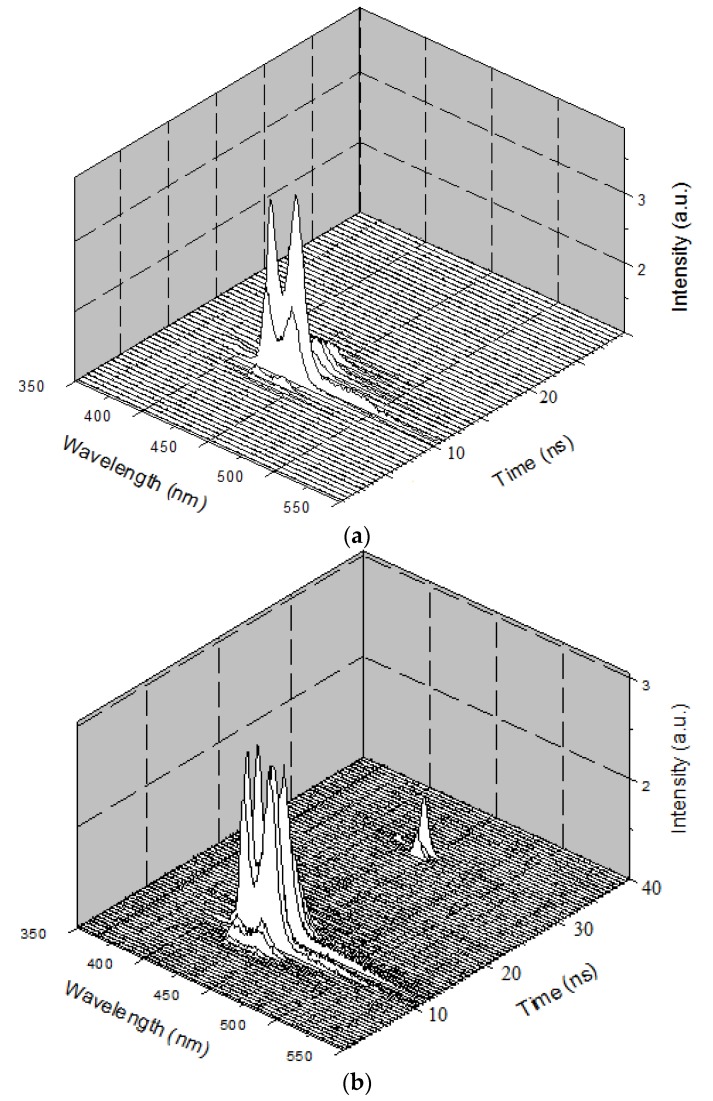

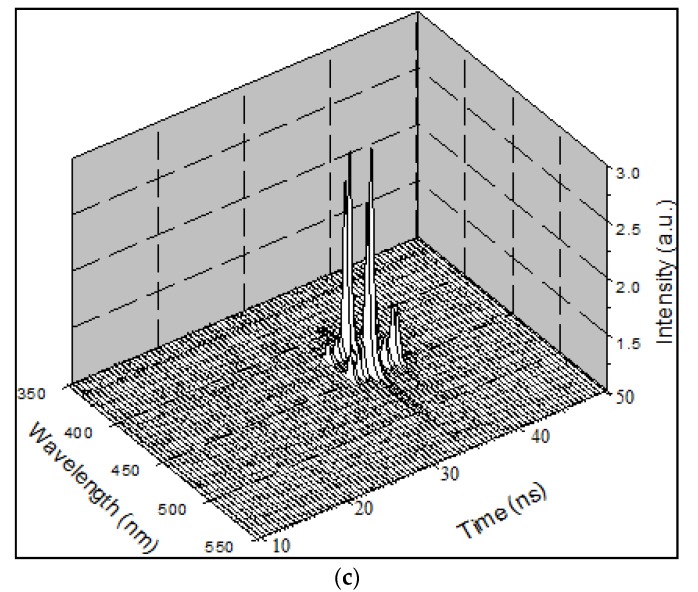
Three-dimensional ASE profiles that show the various dynamics of RO of (**a**) the monomer; (**b**) the excimer at a slow pace; and (**c**) the excimer at a rapid pace.

Relaxation oscillation (RO) is a phenomenon that is a characteristic of few high-power pulsed lasers [25]. It is a manifestation of the dynamic interaction between the abundant population inversion and the photon flux that passes though such excessive inversion Figure 9a shows a representative RO in the monomeric state, and Figure 9b shows that in the excimeric state. Note that the concentration and pump power had been varied by trial and error to obtain the above two different dynamic conditions. Figure 9c shows another example of RO in the excimeric state that occurred in quick succession, indicating the fact that the excimer of the PFO existed much longer than the monomer. For a low concentration, the sample is mostly monomer with a small percentage excimer. Under this condition, when optical pumping was done, population inversion (PI) increased rapidly and the ASE started prominently at 10 ns (see Figure 9a) reached a maximum at 12 ns and drained off by 14 ns. In this short duration of 4 ns, the ASE starts as a monomer at 423 nm, and the excimer became dominant within one nanosecond, but both decayed. This is to be compared with the situation in Figure 9b. In this case, the concentration of the sample and the pump energy both were higher. Hence, PI was significantly higher than in Figure 9a; under this condition, the first pulse existed only for 2 ns, draining off most of the PI and photon flux (due to strong between PI and high photon density); however, another weaker pulse appeared after a lapse of 18 ns due to residual PI and photon density. This has led to bursts of the ASE pulse narrower in spectral width (2 nm at 423 nm and also at 444 nm), and also time (1 ns). Note the secondary pulse occurring due to RO, with 5 ns separation, at a much quicker pace than the 18 ns time lapse condition in Figure 9b.For more details on the relaxation oscillation dynamics refer to [25].

### 3.4. Temperature Dependence of Excimer

**Figure 10:**
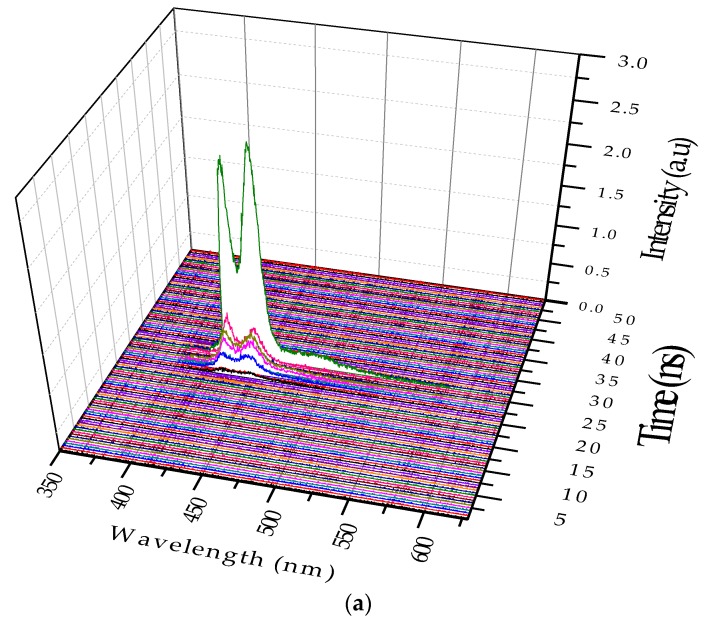

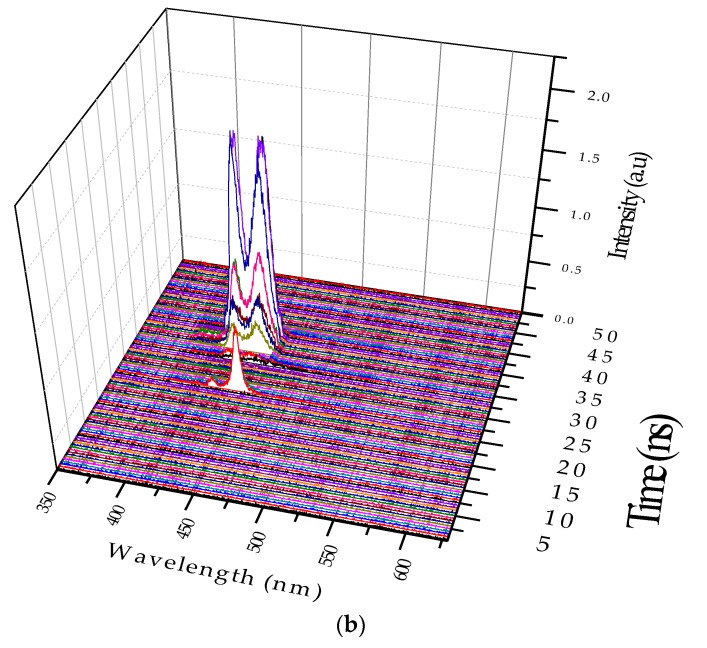
Temperature effect on the excimer and dimer at (**a**) 20 °C and (**b**) 80 °C.

The temporal profile of the excimer formation under two different temperatures is shown in Figure 10, and the conditions are the same as those shown in Figure 7b. The solution concentration was fixed at 0.6 μM, and the pump power was fixed at 8 mJ. As the solution temperature was increased to 80 °C, we could see the disappearance of the LIF at 480 nm (due to the dimer). The ASE intensity at 420 nm (due to the monomer) was relatively stronger than that at 440 nm (due the excimer), i.e., the ratio was $\frac{I_{440}}{I_{420}}$ = 1.3 at 20 °C, but 0.8 at 80 °C, with simultaneous disappearance of the band at 480 nm. Both are due to the thermal agitation that broke the aggregation in the excited state (thus, the excimer became weaker) and in ground state (which made the dimer LIF disappear).

## 4. Conclusions

In this paper, we have reported the spectral and temporal properties of monomer and excimer states of a conjugated polymer PFO under different benzene concentrations and pump rates. This material could produce a laser beam with moderate efficiency at 423 nm for a monomer and 444 nm for the excimer. More importantly, this paper has presented the time evolution (generation, existence, and decay) of the excimer and dimer in a subnanosecond time scale. This report has given a vivid picture of the strong interaction between the excess population inversion and abundant photon flux leading to a burst of photons at 423 and 444 nm, existing for about 500 to 1000 ps. This may be the first and perhaps the only report on picosecond time-resolved spectroscopy of dimers from a conjugated polymer laser.
